# A Microbiological Assessment of Stethoscopes Used by Clinicians in a Tertiary Hospital in Benin City, Nigeria

**DOI:** 10.21315/mjms2023.30.4.9

**Published:** 2023-08-24

**Authors:** Ogie Tada Ehondor, Ephraim Ehidiamen Ibadin

**Affiliations:** 1 Department of Medicine, University of Benin Teaching Hospital, Benin City, Edo State, Nigeria; 2 Medical Microbiology Division, Medical Laboratory Services, University of Benin Teaching Hospital, Benin City, Nigeria

**Keywords:** stethoscope, clinical infection, contamination, pathogen

## Abstract

**Background:**

The hospital environment serves as a niche for pathogenic microorganisms, so efforts are constantly being made to identify the potential mode of microbial pathogen transmission causing clinical infections.

**Objective:**

The aim of this study was to microbiologically examine the stethoscopes used by clinicians at the University of Benin Teaching Hospital (UBTH) in Benin, Nigeria.

**Methods:**

A total of 106 clinicians’ stethoscopes were cleaned using cotton-tipped swabs dampened with normal saline. This included both earpieces along with the diaphragm (three samples per stethoscope). The samples were then sent to the Medical Microbiology Laboratory of UBTH and processed immediately as per the standard guidelines. The emergent colonies were subsequently identified, and antimicrobial susceptibility tests were performed.

**Results:**

A total of 114 (35.8%) bacterial isolates were recovered, including *Staphylococcus aureus* (*S. aureus*) (33.3%), coagulase-negative staphylococci (CoNS) (33.3%), *Bacillus* spp. (22.8%), *Acinetobacter* spp. (5.3%), *Escherichia coli* (*E. coli*) (1.8%) and *Klebsiella* spp. (3.5%). Diaphragms had the highest yield of methicillin-resistant *S. aureus* (MRSA) (46.2%) and CoNS (17.9%). Age (*P* = 0.0387) and cadre of clinician (*P* = 0.0043) were risk factors for contamination, whereas clinicians who never cleaned their stethoscopes (*P* = 0.0044) or cleaned only the earpieces (*P* = 0.0001) had more contaminated stethoscopes.

**Conclusion:**

The contamination rate of stethoscopes used by clinicians in Benin City was 56.6%. There is a need to establish proper stethoscope cleaning practices for all cadres of personnel in clinical practice to minimise health risks to patients and healthcare workers (HCW).

## Introduction

Efforts are being made to explore the role of potential vehicles of microbial pathogens transmission in causing clinical infections as the burden of antimicrobial resistance and treatment failure increases globally (1). Though the most common means of pathogen transfer is via the hands of health professionals and patients, hospital surfaces and equipment also possess a high chance of contamination with hospital pathogens. They may therefore act as reservoirs for microorganisms and when in use, may play a role in transmission (2). Healthcare-associated infection (HAI) is, thus, recognised as a leading cause of increased hospital stay, cost, morbidity and mortality, with poor and middle-income countries being the major contributor to the global burden (2, 3). HAI is defined as an infection occurring in a patient during hospital care or other healthcare facilities that were not manifested or incubated at the time of admission (4). It may even appear after the patient is discharged (4).

The risk factors for HAIs include invasive devices such as intravascular catheters, urinary catheters, central lines, mechanical ventilators and endotracheal tubes (5). They evade the host’s primary defenses and may involuntarily serve as a conduit or vector for microbes to reach the deep-seated tissues and organs to cause infection. There is also discussion of the role of non-invasive medical devices, as poor adherence to infection prevention and control (IPC) practices can cause their contamination, which can lead to the transmission of microbial pathogens to patients and even healthcare workers (HCW) (6).

Stethoscopes are non-invasive devices that are often draped around necks, placed in pockets and frequently touched by unwashed hands day in and out by HCW (6). Even though previous studies have demonstrated colonization or contamination by bacterial pathogens (6–10), little attention has been paid to its potential role in HAI. There is also lack of knowledge among HCWs about its likely role in HAI and a lack of compliance with cleaning guidelines (6, 7, 9). Considering the high patient volume at our facility, this study aimed to microbiologically examine the stethoscopes used by clinicians at the University of Benin Teaching Hospital (UBTH) in Benin.

## Methods

### Study Area

A cross-sectional study was conducted at UBTH in Benin, Nigeria. UBTH is a tertiary health facility with 850 beds and 20 wards that serve Edo State’s healthcare needs and 10 neighbouring states.

### Data Collection and Laboratory Methods

A total of 106 stethoscopes of clinicians were swabbed with cotton-tipped swabs moistened with normal saline. The right and left earpieces and the entire diaphragm (three samples per stethoscope) were swabbed separately, properly labeled, and sent to the Medical Microbiology Laboratory of UBTH. Structured self-administered questionnaires were also issued to clinicians to get information on socio-demography and adherence to cleaning guidelines.

### Sample Processing

The swab samples were inoculated onto Blood and MacConkey agar and streaked. The inoculated agar plates were then incubated at 37 °C for 24 h–48 h. The emergent growth was examined for bacterial identification. Presumptive identification of bacteria was made based on gram stain reaction, colonial characteristics and the requisite biochemical tests as described in standard microbiology texts (11).

### Screening for Methicillin-Resistance

All *Staphylococcus* spp. isolated were analysed for methicillin-resistance by following Clinical and Laboratory Standards Institute (CLSI) guidelines using 30 μg cefoxitin discs (Abtek United Kingdom) (12). The plates were observed after incubation at 35 °C for 18 h. A zone diameter of ≤ 21 mm was considered cefoxitin resistant.

### Extended-Spectrum Beta-Lactamase Detection

The presence of extended-spectrum beta-lactamase (ESBL) was determined in the recovered Gram-negative bacilli using the double disc synergy test and then incubating at 37 °C for 18 h–24 h (13). The ESBL production was inferred as positive if the inhibition zone was expanded between ceftazidime and amoxicillin-clavulanate disc, cefotaxime and amoxicillin-clavulanate disc, or both. *Klebsiella pneumoniae* ATCC 700603 functioned as the positive control strain.

### Disc Susceptibility Test

Disc susceptibility tests were performed on all bacterial isolates using the CLSI guidelines (12). Ofloxacin (5 μg), ciprofloxacin (5 μg), levofloxacin (5 μg), gentamicin (10 μg), erythromycin (5 μg), azithromycin (15 μg) and amoxicillin-clavulanate (30 μg) were used for Gram-positive bacteria. Ofloxacin (5 μg), ciprofloxacin (5 μg), levofloxacin (5 μg), gentamicin (10 μg), ceftazidime (30 μg), ceftriaxone (30 μg), cefotaxime (30 μg) and amoxicillin-clavulanate (30 μg) were used for Gram-negative bacteria. Plates were then incubated overnight at 37 °C for 18 h–24 h. The zone diameter was measured for each antibacterial disc and the susceptibility result was determined using CLSI guidelines.

### Statistical Analysis

The data obtained were analysed with tools like chi-squared using the statistical software INSTAT^®^ (Graph Pad Software Inc., La Jolla, CA, USA).

## Results

A total of 106 medical doctors had their stethoscopes sampled, resulting in 318 parts being sampled. It was found that 114 (35.8%) of these samples were culture-positive, yielding various bacterial isolates. This included 102 Gram-positive bacteria, namely *S. aureus* (33.3%) (methicillin-resistant *S. aureus* [MRSA] [26.3%], methicillin-sensitive *S. aureus* [MSSA] [7.0%]), coagulase-negative staphylococci (33.3%) (methicillin-resistant coagulase-negative staphylococci [MRCoNS] [9.6%], methicillin-sensitive coagulase-negative staphylococci [MSCoNS] [23.7%]), *Bacillus* spp. (22.8%) and 12 Gram-negative bacteria namely *Acinetobacter* spp. (5.3%), *Escherichia coli* (*E. coli*) (1.8%) and *Klebsiella* spp. (3.5%). Diaphragms had the highest yield of MRSA (46.2%) and MRCoNS (17.9%) compared with other stethoscope parts sampled (Table 1).

About 60 stethoscopes (56.6%) were culture-positive. While gender, years of practice and the number of patients seen daily did not significantly affect the contamination rate of stethoscopes (*P* > 0.05), age (*P* = 0.0387) and clinician cadre (*P* = 0.0043) affected contamination rates (Table 2).

According to the study, many medical doctors cleaned their stethoscopes once a day. However, stethoscopes that had never been cleaned (*P* = 0.0044) and those that only the earpiece cleaned had a much higher chance of contamination (*P* = 0.0001). Although > 90% of the doctors admitted sharing stethoscopes with colleagues, this was not a risk factor for contamination (*P* = 0.7602) (Table 3).

Table 4 shows the pattern of antimicrobial susceptibility for Gram-positive bacterial isolates. *Bacillus* spp. exhibited the best susceptibility rates to fluoroquinolones (ciprofloxacin, levofloxacin and ofloxacin) (> 90%) while MRCoNS showed the least susceptibility (< 75%). Methicillin susceptible coagulase-negative staphylococci and MSSA displayed the best susceptibility rates to gentamicin (> 85%). Overall, isolates showed poor susceptibility rates to erythromycin (61.8%), whereas moderate activity was observed against amoxicillin-clavulanate (67.6%).

There was no production of ESBL in the recovered Gram-negative bacteria. Levofloxacin was the most active antibiotic against *Acinetobacter* spp., *E. coli* and *Klebsiella* spp. recovered (91.7%). The cephalosporins exhibited poor activity against bacterial isolates (ceftazidime-50%, cefotaxime-50% and ceftriaxone-50%), while the least active antibiotic was amoxicillin-clavulanate (33.3%) (Table 5).

## Discussion

About 56.6% of the stethoscopes sampled were culture-positive in this study. The contamination rate found is lower than that reported in previous studies conducted in Nigeria, Ethiopia and India, which had 79%, 85.9% and 90%, respectively (7–9). However, it is comparable with another Indian study which showed a 56% contamination rate (10). Stethoscopes get contaminated by the hands of HCW and by contact with patients, their body fluids or their surrounding (7, 8). Nonetheless, this finding indicates poor stethoscope cleaning practices. It is worrisome considering the prevalence of MRSA (17.0%) and MRCoNS (6.6%) recovered from the diaphragms of stethoscopes sampled, as they may serve as the vehicle for pathogen transmission to patients. Recently, MRSA and MRCoNS have been recovered from samples of infected patients in Benin City, Nigeria with 38% of *S. aureus* and 41.5% of CoNS harbouring the *mecA* gene, indicating methicillin resistance (14). The finding of *Klebsiella* and *Acinetobacter* spp. on the diaphragms were also comparable with previous studies (7–9), which further emphasises poor stethoscope disinfection practice.

Similarly, the recovery of MRSA, Enterobacteriales and *Acinetobacter* spp. from the earpiece of stethoscopes is of great concern, especially in an environment where most doctors (> 90%) share stethoscopes. These microorganisms have previously been identified as etiologic agents of otitis media in Benin City, Nigeria (15). Otitis externa has also previously been observed in a nurse following extensive stethoscope use, from which the causal pathogen, *S. aureus*, was also isolated (16). Therefore, stethoscopes could act as a means of bacterial otopathogen transmission among HCW.

Although clinicians’ years of experience did not affect the contamination rate, house officers were likelier to have a higher contamination rate of stethoscopes than other cadres of clinicians. House officers are the lowest cadre of medical doctors, being comprised of clinicians, on a compulsory 1-year training programme in a teaching hospital after the bachelor’s degree. They are, therefore, the least experienced and more likely to be less grounded in standard precautions and IPC practices, including stethoscope disinfection. There may be the need for targeted training of this staff in IPC practices across hospitals where the programme exists, as they are a critical mass in patient care.

Very few clinicians in this study disinfected their stethoscopes after each patient contact, while the majority cleaned their stethoscopes once a day. This finding can be compared with several studies (7–10). The highest contamination rate was found in the stethoscopes that were never disinfected. This finding was significant and unsurprising as there is a higher survival chance and microorganisms transmission in the absence of IPC practices such as stethoscope disinfection. This observation also agrees with previous studies (7, 10, 17).

Similarly, stethoscopes in which only the earpiece was disinfected had the highest contamination rate compared to those who cleaned every part. Guidelines for stethoscope disinfection emphasise cleaning of earpieces and diaphragms (6). Moreover, the diaphragm has a broader surface area and gets in direct contact with the patients; hence, it’s more likely to be contaminated with patients’ transient flora or potential pathogens (17).

Methicillin-resistant staphylococci showed moderately poor susceptibility profiles to commonly available antibacterial drugs, notably fluoroquinolones. Methicillin resistance occurs due to an additional penicillin-binding protein 2a (PBP2a) expression with a low affinity to methicillin and most other β-lactams (14). However, associations between methicillin and increased fluoroquinolone resistance have been reported due to using other antibacterial drugs (18). Similarly, the macrolides (erythromycin and azithromycin) showed poor activity against MRSA and MRCoNS. Macrolide-resistant MRSA has been reported worldwide and is typically characterised by narrow therapeutic options (19). Our finding of poor susceptibility of MRSA and MRCoNS obtained from stethoscopes is similar to some studies and further highlights the risk posed to patients and HCWs when IPC practices are not strictly followed (6, 7, 17).

Although there were no ESBL-producing Gram-negative bacterial isolates, cephalosporins susceptibility profiles were found to be low and the fluoroquinolone-levofloxacin showed the highest activity against the isolates. This finding, however, contrasts with earlier research that indicated that these organisms did not respond well to fluoroquinolones (6, 8, 17). However, the cephalosporins showed poor activity in these studies.

## Conclusion

There was a 56.6% contamination rate among stethoscopes used by clinicians at UBTH, Benin City, Nigeria. To reduce the risk of HAI in patients and HCW, the finding serves as a wake-up call to profoundly embed proper stethoscope cleaning practices across all cadres of personnel in clinical practice.

## Figures and Tables

**Table 1:** Distribution of microorganisms in relation to part of stethoscope sampled

Part of stethoscope	No. culture positive	MRSA	MSSA	MRCoNS	MSCoNS	*Bacillus* spp.	*Klebsiella* spp.	*Acinetobacter* spp.	*E. coli*
Diaphragm (*n* = 106)	39	18 (46.2)	4 (10.3)	7 (17.9)	4 (10.3)	3 (7.7)	2 (5.1)	1 (2.6)	0
Left ear-piece (*n* = 106)	38	6 (15.8)	2 (5.3)	3 (7.9)	13 (24.2)	9 (23.7)	2 (5.3)	4 (10.5)	1 (2.6)
Right ear-piece (*n* = 106)	37	6 (16.2)	2 (5.4)	1 (2.7)	10 (27.0)	14 (37.8)	0	1 (2.7)	1 (2.7)

Total	114	30 (26.3)	8 (7.0)	11 (9.6)	27 (23.7)	26 (22.8)	4 (3.5)	6 (5.3)	2 (1.8)

Notes: MRSA = methicillin-resistant *S. aureus;* MSSA = methicillin sensitive *S. aureus*; MRCoNS = methicillin-resistant coagulase-negative staphylococci; MSCoNS = methicillin sensitive coagulase-negative staphylococci; number in brackets = value in percentages

**Table 2:** Effect of socio-demography on the distribution of microorganisms recovered from stethoscopes

	No. of participants (*n* = 106)	No. culture positive (*n* = 60) *n* (%)	*P*-value
Gender			0.967
Male	47	26 (55.3)	
Female	59	34 (57.6)	
Age (years old)			
25–35	78	39 (50)	0.039
36–45	28	21 (75)	
Cadre			
House officer	35	24 (68.6)	0.004
Medical officer	14	3 (21.4)	
Junior registrar	37	25 (67.6)	
Senior registrar	20	8 (40)	
Years of practice			
0–5	59	30 (50.9)	0.289
6–10	21	12 (57.1)	
11–15	26	18 (69.2)	
No. of patients seen daily			
0–10	49	31 (63.3)	0.438
11–20	49	25 (51)	
≥ 21	8	4 (50)	

Note: Chi-squared test

**Table 3:** Assessment of cleaning practices of stethoscopes used by medical doctors in Benin City, Nigeria

	No. sampled	Culture positive (%)	*P*-value
Frequency of cleaning			0.004
Once daily	41	26 (63.4)	
After each patient	17	4 (23.5)	
Only after body fluid contact	30	15 (50)	
At random	12	9 (75)	
Never	6	6 (100)	
Part of stethoscope disinfected			0.000
Every part	52	21 (40.3)	
Diaphragm only	30	23 (76.7)	
Ear-piece only	18	16 (88.9)	
Sharing stethoscopes with colleagues			0.760
Yes	101	58 (52.3)	
No	5	2 (40)	

Note: Chi-squared test

**Table 4:** Gram-positive bacteria isolated from stethoscopes and their antimicrobial susceptibility pattern

Bacterial isolate	No. tested	OFX	CIP	LVX	CN	E	AZM	AMC
*Bacillus* spp.	26	24 (92.3)	25 (96.1)	25 (96.1)	20 (76.9)	18 (69.2)	17 (65.4)	22 (84.6)
MRSA	30	20 (66.7)	22 (73.3)	21 (70)	22 (73.3)	13 (43.3)	12 (40)	2 (6.7)
MSSA	8	7 (87.5)	8 (100)	8 (100)	7 (87.5)	6 (75)	6 (75)	6 (75)
MRCoNS	11	8 (72.7)	7 (63.6)	7 (63.6)	5 (45.4)	4 (36.4)	5 (45.5)	0
MSCoNS	27	24 (88.9)	25 (92.6)	26 (96.3)	24 (88.9)	22 (81.5)	23 (85.2)	20 (74.1)
Total	102	83 (81.4)	87 (85.3)	87 (85.3)	78 (76.5)	63 (61.8)	63 (61.8)	69 (67.6)

Notes: MRSA = methicillin-resistant *S. aureus*; MSSA = methicillin-sensitive *S. aureus*; MRCoNS = methicillin-resistant coagulase-negative staphylococci; MSCoNS = methicillin-sensitive coagulase-negative staphylococci; OFX = ofloxacin, CIP = ciprofloxacin; LVX = levofloxacin; CN = gentamicin; E = erythromycin; AZM = azithromycin; AMC = amoxicillin-clavulanate; Number in brackets = value in percentage

**Table 5:** Gram-negative bacteria isolated from stethoscopes and their antimicrobial susceptibility pattern

Bacteria	No. tested	OFX	CIP	LVX	CN	CAZ	CRO	CTX	AMC
*E. coli*	2	1 (50)	2 (100)	2 (100)	2 (100)	1 (50)	1 (50)	1 (50)	1 (50)
*Klebsiella* spp.	4	2 (50)	2 (50)	3 (75)	3 (75)	2 (50)	3 (75)	3 (75)	2 (50)
*Acinetobacter* spp.	6	6 (100)	6 (100)	6 (100)	5 (83.3)	3 (50)	2 (33.3)	2 (33.3)	1 (16.7)
Total	12	9 (75)	10 (83.3)	11 (91.7)	10 (83.3)	6 (50)	6 (50)	6 (50)	4 (33.3)

Notes: OFX = ofloxacin; CIP = ciprofloxacin; LVX = levofloxacin; CN = gentamicin; CAZ = ceftazidime; CRP = ceftriaxone; CTX = cefotaxime AMC = amoxicillin-clavulanate; Number in brackets = value in percentage
